# Care Delivery for Children With Epilepsy During the COVID-19 Pandemic: An International Survey of Clinicians

**DOI:** 10.1177/0883073820940189

**Published:** 2020-07-15

**Authors:** Elaine C. Wirrell, Zachary M. Grinspan, Kelly G. Knupp, Yuwu Jiang, Biju Hammeed, John R. Mytinger, Anup D. Patel, Rima Nabbout, Nicola Specchio, J. Helen Cross, Renée A. Shellhaas

**Affiliations:** 1 Divisions of Child and Adolescent Neurology and Epilepsy, Department of Neurology, Mayo Clinic, Rochester, MN, USA; 2 Departments of Population Sciences and Pediatrics, Weill Cornell Medicine, New York, NY, USA; 3 Department of Pediatrics and Neurology, University of Colorado Anschutz Medical Campus, Aurora, CO, USA; 4 Department of Pediatrics, 12465 Peking University First Hospital, Beijing, China; 5 Paediatric Neurosciences, 4956 Great Ormond Street Children’s Hospital, London, United Kingdom; 6 Department of Pediatrics, Division of Pediatric Neurology, Nationwide Children’s Hospital, The Ohio State University, Columbus, OH, USA; 7 Centre de Reference Epilepsies Rares, Department of Pediatric Neurology, 37072 Necker Enfants Malades Hospital, Imagine Institute, Paris Descartes University, Paris, France; 8 Rare and Complex Epilepsy Unit, Department of Neuroscience, Bambino Gesu’ Children’s Hospital, IRCCS, Rome, Italy and Member of European Reference Network EpiCARE; 9 Developmental Neurosciences, UCL NIHR BRC Great Ormond Street Institute of Child Health, London, WC1 N 1EH, & and Member of European Reference Network EpiCARE; 10 Department of Pediatrics (Pediatric Neurology), Michigan Medicine, University of Michigan, Ann Arbor, MI, USA; * These are co-first authors of this article.

**Keywords:** epilepsy, epilepsy surgery, infantile spasms, telemedicine

## Abstract

**Objective:**

To evaluate the effect of the COVID-19 pandemic on global access to care and practice patterns for children with epilepsy.

**Methods:**

We conducted a cross-sectional, online survey of pediatric neurologists across the world affiliated with the International Child Neurology Association, the Chinese Child Neurology Society, the Child Neurology Society, and the Pediatric Epilepsy Research Consortium. Results were analyzed in relation to regional burden of COVID-19 disease.

**Results:**

From April 10 to 24, 2020, a sample of 212 respondents from 49 countries indicated that the COVID-19 pandemic has dramatically changed many aspects of pediatric epilepsy care, with 91.5% reporting changes to outpatient care, 90.6% with reduced access to electroencephalography (EEG), 37.4% with altered management of infantile spasms, 92.3% with restrictions in ketogenic diet initiation, 93.4% with closed or severely limited epilepsy monitoring units, and 91.3% with canceled or limited epilepsy surgery. Telehealth use had increased, with 24.7% seeing patients exclusively via telehealth. Changes in practice were related both to COVID-19 burden and location.

**Conclusions:**

In response to COVID-19, pediatric epilepsy programs have implemented crisis standards of care that include increased telemedicine, decreased EEG use, changes in treatments of infantile spasms, and cessation of epilepsy surgery. The long-term impact of these abrupt changes merit careful study.

The 2019 coronavirus disease (COVID-19) pandemic has resulted in millions of infections and hundreds of thousands of deaths worldwide,^1^ in spite of community mitigation strategies to slow the transmission of disease and protect vulnerable populations.^2^ As a result, the practice of medicine has changed dramatically. Infants and immunocompromised children are at risk for severe infections; however, children overall are less severely affected than adults.^3,4^ Many pediatric healthcare resources have contracted to accommodate critically ill adults, limit exposures to patients and staff, and conserve personal protective equipment. These efforts have disrupted health care delivery to children and led to innovations in care provision, such as expansion of telehealth.

As hospitals became overrun with COVID-19 patients, the risk of visits for care of other disorders began to outweigh the risk of deferred care or alternative approaches. In many centers, other inpatient admissions or surgeries have been limited to life-threatening conditions.

Epilepsy often starts in childhood^5^ and is the second most burdensome neurologic disorder worldwide.^6^ Children with uncontrolled seizures have high rates of psychiatric and cognitive comorbidities and are at risk of injury and death.^7,8^ For children with developmental and epileptic encephalopathies, such as new-onset infantile spasms, delayed or ineffective treatment may permanently worsen developmental trajectories.^9^

We sought to gather a global perspective about the impact of COVID-19 on pediatric epilepsy practice. We developed and administered an English-language online survey to test the hypothesis that the COVID-19 pandemic has changed healthcare delivery to children with epilepsy, as well as to describe these changes.

## Methods

### Study design

We conducted a cross-sectional, online survey of world pediatric neurologists. The sampling frame included members of the International Child Neurology Association (ICNA), the Chinese Child Neurology Society, the Pediatric Epilepsy Research Consortium (PERC), and the Child Neurology Society (CNS). We aimed to assess the experience from a variety of centers, countries, and regions, rather than to account for the practices of all individual members. For example, we expected some countries would have few individual respondents because of the small numbers of pediatric neurologists in some parts of the world. Participants were recruited via an email to all ICNA members and all Chinese Child Neurology Society members, a post on the CNS Connect “Open Forum” message board, and an email to all PERC members.

### Survey Design

The survey covered the following topics: (1) general information about practice; (2) shifts in outpatient clinical activities and access to electroencephalography (EEG); (3) approach to new-onset seizures and infantile spasms; (4) management of children with developmental and epileptic encephalopathies; (5) use of dietary therapies; and (6) use of epilepsy surgery. The questions on dietary therapies were only included in the survey sent to PERC and CNS as most US institutions initiate the ketogenic diet with an admission to the hospital in contrast to some other regions of the world. Items included structured choices as well as free text responses.

### Measures

Several items used semiquantitative measures, asking respondents to estimate numbers and percentages. In addition, one item used a Likert-type scale (1 to 10) to ask clinicians about the perceived degree of social distancing vigilance by families of their patients with developmental and epileptic encephalopathies. Free text questions gathered qualitative data to supplement several answer choices, and to provide anecdotes about clinical experience with COVID-19. These responses were reviewed for common themes, using a modified thematic analysis approach.

### COVID-19 Burden

We downloaded reports of COVID-19 cases and deaths from the *New York Times* ^10^ for US states and from Our World In Data^11^ for countries. We used mortality rate on April 15, 2020, as a rough measure of severity of the pandemic in each of these geographic areas, and subdivided burden into quartiles, based on number of reported deaths per 1 000 000 population, as follows: low (<2.6), medium (2.6-13.8), high (13.8-53.7), and very high (>53.7).

### Missing Data

We report the degree of missing data but did not impute missing values.

### Statistical Methods

Analyses were performed using SPSS, version 26.0 (IBM, Armonk, NY), and R, version 4.0.^12^ To compare categorical variables, we used the chi-square test. To compare continuous variables, we used independent *t* tests, the Wilcoxon test (paired or unpaired), and Spearman correlation, as appropriate. To assess for an association between practice (multinomial) and COVID-19 burden (ordinal), we used a modified Cochrane-Armitage Test.^13^ *P* values less than .05 were considered statistically significant.

## Results

From April 10 to 24, 2020, there were 212 survey respondents, 147 through ICNApedia (from 49 of 123 [40%] countries represented by ICNA), and 65 through the Survey Monkey website sent to PERC and CNS members. Forty-six US pediatric epilepsy centers and 64 US child neurology centers were represented. The 46 pediatric epilepsy centers represent 32% of the estimated 144 US pediatric epilepsy centers.^14^

### Demographic Data

Respondents included individuals from 6 continents, most from Asia (40.6%) and North America (36.8%) (Table 1). Most respondents (58.8%) were general child neurologists; however, an important minority (34.6%) were pediatric epileptologists. Significantly more respondents from North America identified as pediatric epileptologists, compared to other regions (60% vs 20%; *P* < .001). Of 73 pediatric epileptologists, 54 (74.0%) spent part of their clinical duties in an epilepsy monitoring unit.

**Table 1.** Demographic Data of 212 Child Neurologists Who Responded to an Online Survey Regarding the Impact of the COVID-19 Pandemic on Clinical Practice for Pediatric Epilepsy.

Continent	Countries represented (number from each country)^a^	Practice type
Asia (N = 86)	China (53), India (8), Japan (4), Indonesia (3), Kuwait (2), Myanmar (2), Pakistan (2), Philippines (2), Iran (2), Iraq (1), Azerbaijan (1), Kazakhstan (1), Israel (1), Saudi Arabia (1), Taiwan (1), Thailand (1), United Arab Emirates (1)	General child neurologist (65) Pediatric epileptologist (18) Other subspecialty child neurologists (2) Data missing (1)
North America (N = 78)	United States (71), Jamaica (2), Canada (1), Columbia (1), Costa Rica (1), Mexico (1), Trinidad and Tobago (1)	General child neurologist (27) Pediatric epileptologist (46) Other subspecialty child neurologists (5)
Europe (N = 17)	France (2), Greece (2), Netherlands (2), United Kingdom (2), Belgium (1), Croatia (1), Denmark (1), Georgia (1), Hungary (1), Italy (1), Romania (1), Russian Federation (1), Spain (1)	General child neurologist (10) Pediatric epileptologist (4) Other subspecialty child neurologists (2) General pediatrician doing neurology (1)
South America (N = 15)	Brazil (11), Peru (2), Argentina (1), Ecuador (1)	General child neurologist (11) Pediatric epileptologist (2) Other subspecialty child neurologists (2)
Africa (N = 11)	Nigeria (3), Egypt (2), Tunisia (2), Zimbabwe (2), Kenya (1), South Africa (1)	General child neurologist (6) General neurologist (1) Pediatric epileptologist (2) Other subspecialty child neurologists (1) General pediatrician doing neurology (1)
Oceania (N = 4)	Australia (3), New Zealand (1)	General child neurologist (3) Pediatric epileptologist (1)

^a^1 respondent did not provide location, but identified as a general child neurologist.

Of the 210 respondents who identified their country of origin (or if from the US, their state of origin), the COVID-19 burden was very high for 21.4%, high for 16.7%, medium for 42.9%, and low for 19%. Respondents from the United States and Europe had higher COVID-19 burdens than those from other continents (*P* < .001).

### How Did COVID Change Clinical Practice?

#### a. Outpatient Clinical Activities

Prior to COVID-19, respondents reported a median proportion of clinical time spent in outpatient clinical activities of 60% (interquartile range [IQR] 40, 80). The median inpatient clinical time was 40% (IQR 20, 60).

**Figure 1.**
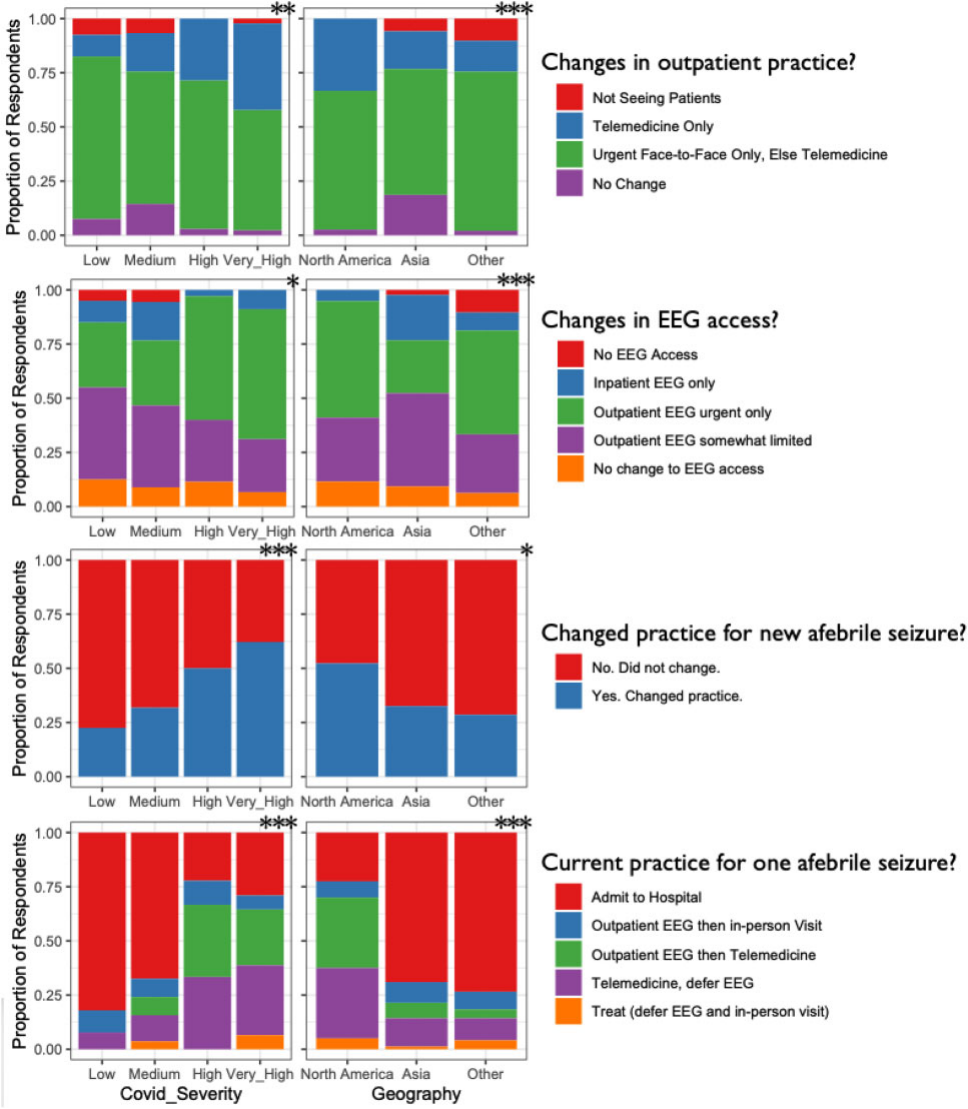
Changes in practice (rows) were related both to regional COVID-19 burden (left column) and the location of practice (right column). All panels illustrate a statistically significant difference (*P < .05, **P < .01, ***P < .001; left column modified Cochrane Armitage test, right column chi-square test).

As a result of COVID-19, nearly all (91.5%) reported markedly reduced face-to-face visits and increased use of telemedicine (Table 2). Restriction of outpatient face-to-face visits was associated with higher COVID-19 burden (*P* < .01) and with geographical region (*P* < .0001; Figure 1).

**Table 2.** Impact of COVID-19 on Various Aspects of Practice.

Area of practice	Percentage reporting impact on usual practice due to COVID-19	Details
Outpatient clinical activities (n = 212)	91.5%	5.2% were unable to see any outpatients 24.7% saw outpatients exclusively by telemedicine 70.1% saw most outpatients by telemedicine but still saw urgent cases face-to-face
Access to EEG (n = 212)	90.6%	3.6% had no access to EEG 13.5% could access only inpatient EEG 44.8% could access outpatient EEG only for urgent cases 38.0% had more limited EEG access but could still obtain studies for most cases
Children with new-onset seizures (n = 199)	38.2%	For a new *single* afebrile seizure (n = 74): 5.4% recommended hospital admission 20.3% recommended EEG followed by face-to-face consultation 27.0% recommended EEG followed by telemedicine consultation 39.2% recommended telemedicine consultation prior to deciding on need for EEG 1.4% recommended face-to-face consultation prior to deciding on need for EEG 6.8% recommended starting ASMs and deferring both EEG and consultation For new-onset *recurrent* afebrile seizures (n = 76): 25.0% recommended hospital admission 18.4% recommended EEG followed by face-to-face consultation 28.9% recommended EEG followed by telemedicine consultation 21.1% recommended telemedicine consultation prior to deciding on need for EEG 6.6% recommended starting ASMs and deferring both EEG and consultation
New-onset infantile spasms (n = 203)	37.4%	Lower likelihood of hospital admission: Pre-COVID-19: median admission rate 75% (IQR 5, 100) Since pandemic: median admission rate 5% (IQR 0, 50) Lower likelihood of ACTH and higher likelihood of oral steroids and nonstandard therapies (see Figure 1)
Dietary therapies^a^ (n = 39)	92.3%	Inpatient ketogenic diet initiation (n = 39) 30.8% could not initiate inpatient dietary therapy for any child 61.5% could initiate inpatient dietary therapy only for urgent cases 7.7% could initiate inpatient dietary therapy for any child Outpatient ketogenic diet initiation (n = 34) 26.5% could not initiate outpatient dietary therapy for any child 52.9% could initiate outpatient dietary therapy by telemedicine only for urgent cases 17.6% could initiate outpatient dietary therapy by face-to-face consultation only for urgent cases 2.9% could initiation outpatient dietary therapy for any child Follow-up visits for children on dietary therapy (n = 37) 51.4% done by telemedicine only 5.4% reduced frequency of visits due to pandemic 43.2% reported no change in how follow-up visits were done Ketogenic surveillance labs (n = 37) 45.9% reduced frequency of surveillance labs during the pandemic 54.1% reported no change in labs (but one sent sample collection kit by mail to families)
Epilepsy surgery^b^ (n = 46)	97.8%	Admission for epilepsy surgery evaluation 54.3% unable to admit any child 39.1% admissions restricted to urgent/life-threatening cases only 4.3% mild reduction in admissions 2.2% no impact Epilepsy surgery for cases worked up before the pandemic 34.8% unable to perform any epilepsy surgery 56.5% can perform epilepsy surgery only for urgent/life-threatening cases 2.2% mild reduction in epilepsy surgery access 6.5% no impact

Abbreviations: ACTH, adrenocorticotropic hormone; ASMs, antiseizure medications; EEG, electroencephalography; IQR, interquartile range.

^a^ Responses limited to Pediatric Epilepsy Research Consortium (PERC) and the Child Neurology Society (CNS) members.

^b^ Responses limited to pediatric epileptologists.

#### a. Access to EEG

Nearly all (90.6%) reported reduced access to EEG as a result of COVID-19, with 3.6% reporting no EEG access and 13.5% reporting inpatient access only (Table 2). Limitations in access to EEG were associated with COVID-19 burden (*P* < .01) and with geography (*P* < .0001; Figure 1).

#### a. Approach to children with new-onset seizures

Among the 199 respondents who described their clinical approach to children with new-onset seizures, 76 (38.2%) reported a change since COVID-19. Change in practice was associated with COVID-19 burden (*P* < .0001) and with geographical region (*P* < .05; Figure 1).

Of the 74 who reported a practice change and provided data regarding their current practice for children presenting with a *single afebrile seizure* (Table 2), 5.4% would recommend hospital admission. Of the 70 who would opt for outpatient workup, only half would routinely order an EEG prior to consultation and 70% would perform the outpatient telemedicine consultation.

Of the 76 describing their current approach to children with *new-onset, recurrent afebrile seizures* (Table 2), 25.0% would recommend hospital admission. Of the 57 opting for outpatient assessment, most (63.2%) would obtain a routine EEG prior to consultation and most would perform the consultation using telemedicine (66.7%).

For the 177 who provided additional details on their approach to a first unprovoked seizure, evaluation of a single afebrile seizure was associated both with COVID-19 burden (*P* < .0001) and geography (*P* < .05). In particular, the likelihood of an admission was much lower in North America (22%) than in other regions (Asia 67% and Other 73%; Figure 1). The approach to new-onset recurrent afebrile seizures did not significantly vary by COVID-19 burden (*P* = .4) nor by geography (*P* = .19).

#### a. Approach to infantile spasms

**Figure 2.**
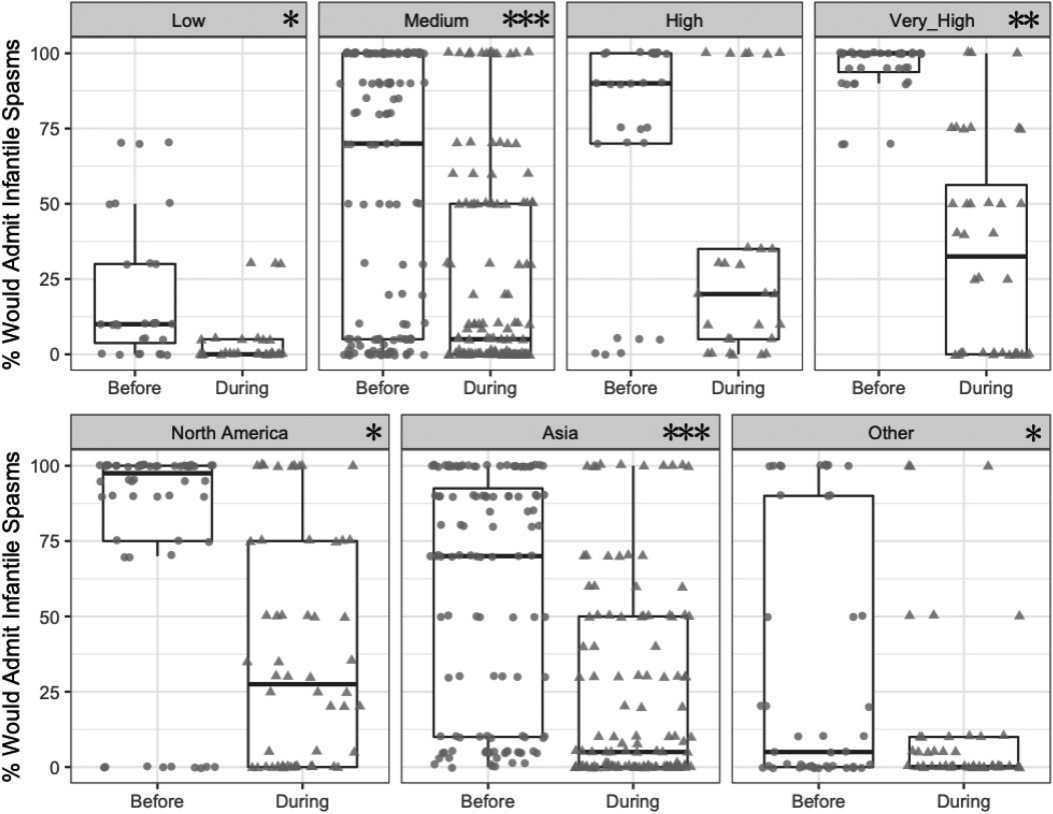
Estimated percentage of children with new-onset infantile spasms admitted to the hospital before (circles) and during (triangles) the COVID-19 pandemic, stratified by COVID-19 burden (top row) and by geography (bottom row). Analyses are based on 68 respondents who had changed their practices. Points represent individual respondent estimates; boxes show 25th, 50th, and 75th percentiles; whiskers estimate 95% confidence intervals. Stars in each panel indicate a significant shift within the displayed subgroup. (*P < .05, **P < .01, ***P < .001; Wilcoxon paired test).

Of the 203 respondents who described their intended or actual practice to diagnose and treat infantile spasms, 76 (37.4%) indicated that the COVID-19 pandemic had resulted in a practice change (Table 2). Of 68 who estimated the likelihood of admission, 69% were less likely to admit, at all levels of COVID-19 severity and across geographies. The reduced likelihood of admission was larger in areas with higher COVID-19 severity (Spearman rho = 0.35, *P* < .001; Figure 2).

Most (75.7%) indicated they would be comfortable diagnosing infantile spasms based on an interictal outpatient EEG with recorded sleep combined with a home video of a typical event. In the situation where EEG was not rapidly available, just more than half (54.1%) agreed that a diagnosis could rest on a characteristic video of infantile spasms obtained by the family. However, many respondents indicated in their comments that diagnosis without EEG would require high-quality video, highly characteristic semiology, and known risk factors for infantile spasms. Some expressed a willingness to diagnosis by video alone because of worsening developmental outcomes with treatment delay. Other respondents indicated reluctance to diagnose by video, because other movements may mimic infantile spasms and misdiagnosis of children without infantile spasms could result in inappropriate exposure to the side effects of treatment.

**Figure 3.**
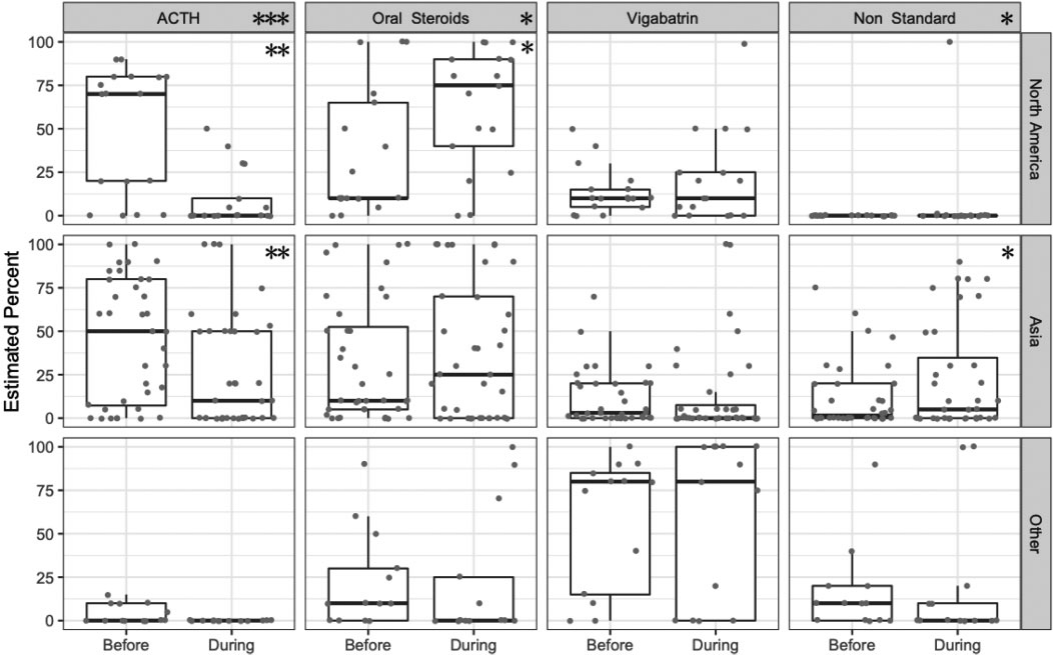
Estimated percentage use of 4 medications (columns) for infantile spasms in 3 geographies (rows; North America [n = 17], Asia [n = 36], and other continents [n = 13]) before and during the COVID-19 pandemic. Gray dots represent the estimated percent of prescription by individual respondents; boxes show 25th, 50th, and 75th percentile; whiskers estimate 95% confidence intervals. Stars in the column headings indicate a significant global shift in estimated use of that medication. Stars in each panel indicate a significant shift in the specified geography. (*P < .05, **P < .01, ***P < .001; Wilcoxon-paired test).

Sixty-six respondents estimated their percentage of use of various treatments for infantile spasms before and during the pandemic, including 17 from North America, 36 from Asia, and 13 from other continents (Figure 3). With the pandemic, there was a global shift away from adrenocorticotropic hormone (ACTH) (median estimated percentage 20% [IQR 0, 75] to 0 [0, 28]; *P* < .001) and toward oral steroids (10% [5, 50] to 28% [0, 80]; *P* < .05). Estimated vigabatrin use was roughly stable, (10% [0, 29] to 5% [0, 38]; *P* = .2), though there were notable geographic variations in how often it was prescribed (before pandemic North America and Asia 5% [0, 20] vs other continents 80% [15, 85]; *P* < .001). Estimated use of non-tandard medications (treatments other than ACTH, oral steroid or vigabatrin) was rare in North America (one respondent) but increased in Asia (from 1.5% [0, 20] prepandemic to 5% [0, 35] during the pandemic; *P* < .05). Higher COVID-19 burden correlated with greater use of oral steroids (*P* = .002) and vigabatrin (*P* = .005) but did not correlate with reduced use of ACTH (*P* = .39) or greater use of non-standard first-line agents (*P* = .09).

Sixty-three respondents indicated how they would manage an infant with newly diagnosed infantile spasms who had been exposed to the novel coronavirus. Most (69.8%) would choose a standard first-line infantile spasms treatment—usually vigabatrin (27/63) or oral steroids (14/63). Importantly, 17 (27.0%) would choose a non-standard option, and 2 (3.2%) would defer infantile spasms treatment until the infant’s COVID-19 infection status was known.

One hundred seventeen respondents reported seeing at least 1 child with newly diagnosed infantile spasms since onset of the COVID-19 pandemic [median number of cases 2 (IQR 1, 5)].

Thirty-five respondents reported treating at least 1 child with hormonal therapy during the pandemic. Of these, 9 reported that adverse effects due to therapy had occurred (none related to infection), but only 1 child required a change to vigabatrin.

#### a. Children with developmental and epileptic encephalopathies (DEEs)

Regardless of location, respondents noted that most families of children with DEEs were very cautious regarding social distancing, ranking their degree of caution at a mean of 7.94/10 (SD 2.0) on a Likert scale. Only 11 of 185 respondents reported having a patient with DEE who developed COVID-19 infection (7 from Asia, 2 North America, 1 Africa, 1 South America). There was a trend for respondents who reported having a DEE patient with COVID to report a perception of decreased vigilance of social distancing in their patient population (*P* = .055). Four of these respondents provided further details. In 2 children, seizures worsened. Both were managed at home. One child with DEE required ICU admission for respiratory distress, but not for worsening seizures. The fourth child had no worsening of seizures and did not require admission for COVID-19 symptoms.

#### a. Provision of dietary therapies for epilepsy

Questions about dietary therapies for epilepsy were only posed to the PERC and CNS members. Among 48 who worked in a center that routinely offered ketogenic diet therapy, 37 provided details of their prepandemic dietary therapy practices. The proportions of children (median, IQR) initiated on the various dietary options were as follows: classical ketogenic diet in the inpatient setting (70%, 45, 88), classical diet in the outpatient setting (0%, 0, 20), modified Atkins diet (10%, 5, 30), and low glycemic index diet (0%, 0,1).

Inpatient practice had changed since the COVID-19 pandemic (39 respondents; Table 2). Inpatient dietary initiation was very limited or not possible for more than 92% of respondents. Decreased inpatient access to the ketogenic diet was not associated with COVID-19 burden (*P* = .70).

Of the 34 respondents who provided details on outpatient dietary therapy practice changes, 26.5% were unable to offer outpatient diet initiation to any child, and 70.5% could only start the diet as an outpatient in urgent cases (Table 2). Almost all respondents (37/38) reported that their ketogenic dieticians were able to do telemedicine visits.

Regarding follow-up of children on dietary therapy, 51.4% offered follow-up via telemedicine only and 5.4% reported that follow-up visits were delayed until the pandemic stabilizes. The frequency of obtaining surveillance labs was reduced in 45.9% of cases.

#### a. Access to epilepsy surgery

For questions on epilepsy surgery, 46 of 73 pediatric epilepsy specialists responded. More than half were unable to admit *any* child for presurgical assessment, and more than one-third reported that *all* epilepsy surgeries were on hold because of COVID-19 (Table 2). Of the remainder, access to both the epilepsy monitoring unit or epilepsy surgery was generally limited to urgent or life-threatening cases only.

### Neurologic Complications of Children With COVID-19 Who Did Not Have Preexisting Epilepsy

Forty-eight respondents reported having seen at least 1 child without preexisting epilepsy who presented with COVID-19. Twenty-nine percent of respondents from Asia and 17% of respondents from North America reported seeing at least 1 case. The median number of individual children seen by each of these clinicians was 2 (IQR 1, 5), for a total of 160 children with COVID-19. Seventeen of these children (10.6%) were reported to have had acute symptomatic or febrile seizures. Encephalopathy was present in 7, and 7 had other neurologic symptoms (headache, weakness, and muscle pain). Seven children had anosmia, though respondents indicated in their free-text replies that this number may be an underestimate as this symptom is difficult to diagnose in young children.

## Discussion

### Summary of Key Findings

Our international survey, which included >200 respondents from 49 countries, found that clinical care for children with epilepsy has changed dramatically in response to the COVID-19 pandemic. Areas with higher COVID-19 burden had more practice changes, such as greater restriction in outpatient face-to-face visits, decreased use of EEG in children with their first afebrile seizure, decreased admission rate for new-onset infantile spasms, and increased shift toward use of oral treatments for infantile spasms. However, restricted access to inpatient ketogenic diet initiation and inability to admit children for presurgical assessment or to perform epilepsy surgery were reported from nearly all centers, regardless of COVID-19 burden. Additionally, although acute symptomatic seizures were not reported in adults,^15^ our respondents suggest that acute symptomatic or febrile seizures do occur in children with COVID-19.

#### Infantile spasms

We were concerned to find an increase in use of nonstandard treatments for new-onset infantile spasms. Several rigorous studies have clearly demonstrated that these have lower efficacy than standard agents^9,16^ and that delaying effective therapy leads to a permanent decrease in developmental potential.^17^ High-dose oral steroids, with or without combination vigabatrin, have seen shown to be efficacious for infantile spasms^18,19^ and can be rapidly started if inpatient hospitalization is not possible. *We therefore strongly encourage all clinicians who care for children with infantile spasms to use standard first-line treatment (ACTH, prednisolone, or vigabatrin).* Although there may be concerns regarding the use of high-dose oral steroid or ACTH in children with potential COVID-19 exposure, vigabatrin should be considered in such cases as opposed to nonstandard therapies or delaying treatment. We were also concerned about the implications of reduced access to EEG, and the potential delays in diagnosis and treatment. Timely diagnosis and effective therapy are critical to maximize developmental outcomes.^9,17^ *We encourage pediatric neurology programs to prioritize evaluation of suspected infantile spasms*. Of interest, our survey was administered 1 week after the CNS issued a consensus recommendation for use of oral steroids, as opposed to ACTH, as a crisis standard of care for new-onset infantile spasms—this may have influenced the response from child neurologists in North America. These recommendations are now published in summary^20^ and in full. The variations in care suggest there is an opportunity for international dissemination of guidelines^21-23^ and quality measures^24^ for infantile spasms care.

#### Epilepsy Surgery

It is strongly recommended that persons with focal epilepsy whose seizures remain uncontrolled after trials of 2 antiseizure medications be considered for surgical evaluation to determine whether they may be candidates for resection.^25^ In the developing brain, the negative impact of poorly controlled seizures on cognition is significant.^26^ For appropriately selected cases, early surgery improves long-term developmental outcomes, behavior, and quality of life.^27,28^ Additionally, uncontrolled convulsions increase the risk for sudden unexpected death in epilepsy (SUDEP).^7^ In young children with frequent ongoing seizures due to a surgically treatable structural brain abnormality, the risk-benefit of delayed surgery should be carefully considered. World-wide, delays in epilepsy surgery evaluations and resections raise the sobering possibility that a cohort of children will suffer irreparable neurodevelopmental harm or even death as a consequence of the COVID-19 pandemic.

#### Ketogenic diet

The ketogenic diet can markedly reduce seizure burden, improve alertness, and improve development in children with drug-resistant epilepsy who are not candidates for epilepsy surgery.^29-31^ Less stringent forms of the ketogenic diet, such as the modified Atkins diet or low glycemic index diet, may have similar efficacy for older children; however, the classical ketogenic diet has better efficacy for children under age 2 years.^32^ For young children, inpatient dietary initiation is recommended to manage the risks of hypoglycemia and acidosis.^33,34^ Our survey indicated that access to inpatient care for ketogenic diet initiation has been sharply restricted; as a result, children who require dietary therapies are at risk of either being prescribed a potentially less-effective option (modified Atkins diet or low glycemic index diet rather than classical ketogenic diet) or of suffering important adverse effects while treated at home. Return to prepandemic standards of care should be a priority as epilepsy programs resume clinical operations.

#### Telemedicine

The COVID-19 pandemic has led to a dramatic increase in the use of telemedicine across many specialties. Although this has preserved access to care for families who have reliable Internet and telephone access, there are immediate concerns about disparities in access to care. Several key aspects of the neurologic examination cannot be performed remotely, such a Woods lamp examination to assess for hypopigmented macules, a funduscopic exam, accurate head circumference, or assessment of muscle tone. Furthermore, the impact of telehealth on difficult conversations, such as new diagnoses and sudden unexpected death in epilepsy, is not known.

#### Access to EEG

Nearly all respondents reported reduced access to outpatient EEG, a core investigation in the diagnosis and management of epilepsy. Many respondents were relying on clinical history alone and/or review of home video to make diagnoses of first seizures or new-onset epilepsy, and in some cases, serious epileptic encephalopathies such as infantile spasms. The EEG regularly provides important information regarding epilepsy type and syndrome, which is crucial for planning further investigation and selecting optimal treatment. Furthermore, the EEG may capture subtle seizures that are missed by families. Decision making without EEG may lead to suboptimal treatment strategies and potentially an increase in adverse outcomes.

### Limitations

Because of time constraints and a desire to obtain actionable information given the rapidly shifting nature of the pandemic, there was only a single request to complete the survey. We aimed for a broad sample that represented as many countries and as many US programs as possible at a particular time point rather than providing repeated reminders to increase response rates of individual clinicians. We analyzed the results in the context of publicly reported burden of mortality related to COVID-19 infections but acknowledge that there are regional differences in ability to report cases. Although our target population was the global community of child neurologists, the survey sample was limited to members of the target professional organizations. We did not differentiate between responses from free-standing children’s hospitals versus pediatric care embedded in larger hospitals that also provide care to adults, though we acknowledge that hospital structure likely impacts access to care. Our study also has not looked at the economic status of respondent countries, which may impact their response to such a pandemic. Our survey items focused on changes in practice but did not fully assess the state of practice and access to resources prior to the pandemic. Finally, although the data reflect practice changes reported for a particular week in April 2020, they remain relevant given the likelihood of additional waves of infection.

## Conclusion

Solutions to the range of challenging issues faced by epilepsy care teams and their patients will necessarily vary by geography, national and local resources, health insurance paradigms, and hospital structures. Yet, our data are clear—the COVID-19 pandemic has caused profound changes to the care of children with epilepsy. Given the unknown timing of an effective vaccine and duration of increased burden of COVID-19 on the health care system, and the negative consequences of ongoing seizures, implementation of temporary, crisis standards of care for children with epilepsy are paramount. At the same time, key issues for the coming months and years include prioritization of a global backlog of children who require epilepsy surgery; optimizing access to EEG; and return to evidence-based treatment when crisis standards of care pose higher risk than exposure to the novel coronavirus. Importantly, re-emergence of face-to-face clinical care may be frightening both to families and to clinical care teams. A concerted effort to minimize risk and, as appropriate, maximize reassurance about infection control practices must be implemented.
